# *Helicobacter pylori* Eradication Therapy: Current Availabilities

**DOI:** 10.5402/2012/186734

**Published:** 2012-07-29

**Authors:** M. Gasparetto, M. Pescarin, G. Guariso

**Affiliations:** Unit of Gastroenterology, Digestive Endoscopy, Hepatology and Care of The Child with Liver Transplantation, Department of Pediatrics, University Hospital of Padova, Via Giustiniani 2, 35128 Padova, Italy

## Abstract

*Background*. Though *Helicobacter pylori* (HP) infections have progressively declined throughout most of the industrialized countries, a gradual increase in failure of HP eradication treatments is observed. *Aim*. To critically review evidence on the efficacy of the therapeutic availabilities for HP eradication, as yet. *Methods*. A selection of Clinical Trials, Systematic Reviews and Meta-analyses within the time period 2010–2012, was performed through a Medline search. Previous references were included when basically supporting the first selection. *Results*. An increasing rise in HP resistance rates for antimicrobial agents is currently observed. Further causes of HP treatment failure include polymorphisms of the CYP 2C19, an increased body mass index (BMI), smoking, poor compliance and re-infections. Alternative recent approaches to standard triple therapy have been attempted to increase the eradication rate, including bismuth-containing quadruple therapy, non-bismuth containing quadruple therapy, sequential therapy and levofloxacin-containing regimens. *Conclusions*. The main current aims should be the maintenance of a high eradication rate (>85%) of HP and the prevention of any increase in antimicrobial resistance. In the next future, the perspective of a tailored therapy could optimize eradication regimens within the different countries.

## 1. Introduction

*Helicobacter pylori* (HP) is an ubiquitous gram-negative bacterium. Its specific microbiological features allow its survival in the stomach and the colonisation of the antrum. HP expresses high levels of urease enzyme, producing NH3 and CO_2_ from the urea being present in the gastric juice [1]. This mechanism called acid acclimation regulates the cytoplasmic pH of the bacterium and guarantees the maintenance of a periplasmic pH near neutrality, despite the acidic environment [2]. Once HP has colonised the stomach, an immune response begins; as a result, normal acid secretion and gastric architecture are modified. Consequences are gastritis, peptic ulcer disease, functional dyspepsia, and cancer. HP was first identified in 1982 and by 1989 had been associated with gastric inflammation and ulcers in adults and children [3]. During the 1990s evidence emerged of its etiologic role in stomach cancers in adults. HP was classified in 1994 among the group I carcinogens by the International Agency for Research on Cancer [4]. There are also a lot of associations between HP and other extra intestinal diseases such as cardiovascular diseases, diabetes mellitus, lung diseases, hematologic diseases, neurological diseases, and glaucoma. Nevertheless, more studies are necessary to delineate the cause-effect relationship for any of these issues [5]. *Helicobacter pylori* infects approximately 50% of the adult population [1, 6]: only 20% of HP-infected people are symptomatic whereas 80% of them are symptom-free. 

## 2. Aim and Methods

This paper is conceived to critically review evidence on the efficacy of the therapeutic availabilities for HP eradication, available as yet. A Medline search was performed indicating as keywords “Helicobacter,” “Eradication Regimens,” “Eradication Rate,” “Epidemiology,” “Drug Resistance.” A selection of relevant English-language clinical trials, systematic reviews, and meta-analyses within the time period 2010–2012 was performed. Previous references were included when basically supporting the first selection. PubMed, Cochrane Library, Trip Database, and Ovid Medicine were used as referral databases.

Jadad Scale (score 0 to 5) was used as a predetermined strategy of inclusion for clinical trials.

## 3. Epidemiology of *H. pylori* Infection

Over recent decades, HP infections have progressively declined throughout most of the industrialized countries. HP still represents, by the way, a clinical issue among those people who were infected as children at a time when HP was endemic or among immigrants coming from endemic countries [14, 15]. Many different factors play a role in the development of HP infection, including geography, ethnicity, age, and socioeconomic elements [15]. The progressive decline in infection rates has been secondary to the improvements in sanitation as well as to the use of combination of anti-HP regimens over the past decades. 

At the same time, a gradual increase in failure of HP eradication treatments is found [16] and the task of treating HP has now become increasingly challenging: a fall in the eradication success with standard triple therapy from over 90% in the 1990s to <60% is observed nowadays [17]. The main reason for eradication failures is antimicrobial resistance, in particular clarithromycin (CAM) resistance relates to a reduction in the success of standard triple therapies by 35–60% [18]. 

## 4. *Helicobacter pylori*: Drug Resistance

Since resistance rates for antimicrobial agents rise with their indiscriminate use, clarithromycin (CAM) resistance may be due to the widespread use of this agent for upper respiratory tract infections [19, 20]. De Francesco et al. showed [21] that HP antibiotic resistance rates all over the world are 17.2% for CAM, 26.7% for metronidazole (MNZ), 11.2% for amoxicillin, and 16.2% for levofloxacin. It is known that bacteria can develop efflux channels for CAM, which rapidly transfer the drug out of the bacterial cell, preventing the binding of the antibiotic to the ribosome [22]. It has been speculated that the disruption of the cell wall caused by amoxicillin prevents the development of efflux channels by damaging the wall of the bacterium, thus improving the efficacy of CAM in a second phase of treatment [23]. It has, however, to be taken into consideration that the improved effect with sequential and concomitant therapies compared with standard therapy may also be due to the larger number of antibiotics than to the sequential administration itself [24]. Since one meta-analysis reported an almost 60% decline in eradication rates with standard triple therapy if CAM resistance was present [25], the use of standard triple therapy has been recommended only in those areas where CAM resistance is lower than 15–20%. As a general rule, clinicians should prescribe therapeutic regimens that have a ≥90% efficacy [26]. There are several reasons causing treatment failure, the antimicrobial resistance being the most important; other lower causes are polymorphisms of the CYP 2C19, an increased body mass index (BMI), smoking, poor compliance, and reinfections [8, 27–29]. Since all the different HP therapies are long and complicated, a poor compliance is often found as the real reason of a treatment failure, particularly in the developed countries. New strategies to improve compliance should therefore be applied [30]. 

The prevalence of antimicrobial resistance might be significantly different between countries, which will greatly affect eradication rates [1, 31]. For example, a significant increasing rate of resistance to CAM and MNZ in some parts of Asia including Japan [32] determines a reduced efficacy of PPI-based triple therapy. Cases of Nitroimidazole resistance as well as dual resistance (to both CAM and MNZ) are described [33]. For these latter patients, concomitant therapy may be more suitable than sequential therapy. The increase in MNZ resistance appears, however, less important with respect to CAM, as a dose increase and a longer treatment period consent to avoid HP infection. Given the described geographical variability, the development of better treatments for HP infection should take into consideration the distribution of antibiotic resistance among affected populations in different geographical areas; however, these data are still lacking outside the USA. A recent meta-analysis of five randomized controlled trials totalling 701 patients [34] showed that culture-guided triple therapy is superior to standard triple therapy referring to a higher eradication rate from intention-to-treat analyses (CI 0.77–0.9, *P* < 0.00001) and a lower overall cost. Antimicrobial susceptibility testing is therefore recommended before first-line treatment for HP infection. Another important factor is polymorphism of the CYP2C19 isoenzyme [35, 36] that is responsible for the biotransformation of several PPIs: significant differences in eradication rates have been observed between homozygous and heterozygous extensive metabolizers and poor metabolizers of PPIs (omeprazole being the most affected, followed by lansoprazole). As a consequence, the use of higher doses may be required. Orientals, for instance, are low metabolizers than Caucasians. The influence of age on the efficacy of concomitant treatment is unclear; the only study that has evaluated concomitant therapy in children showed favorable results (94% of eradications in 33 children) [37]. Gender was not found to be a significantly predictive factor for success or failure of HP eradication in patients treated with concomitant therapy [38]. The main current effort should be to maintain the eradication rate of HP above 85% and to prevent any increase in antimicrobial resistance in the general population. 

The possibility of a tailored therapy will be a chance in the next future to optimize the most effective eradication regimen within the various geographical areas; for this proposal, the determination of HP genotype from gastric tissue samples or stools as well as the determination of CYP2C19 polymorphism is required [39].

## 5. Therapeutic Approaches Attempted for Eradication of *H. pylori*

The most widely recommended treatment in international guidelines for the eradication of HP is the Standard Triple Therapy based on a proton-pump-inhibitor (PPI) to which two antibiotics (CAM plus amoxicillin (AMPC or MNZ) are associated for 7 days [37–41]. With few exceptions, the standard triple therapy now provides unacceptably low treatment success (i.e., 80% or less) [42]: two recent double-blind multicentre studies have evidenced low eradication rates with standard therapy (77%)[43, 44] and two meta-analyses on more than 53.000 patients have confirmed this data [45, 46]. As a consequence, new alternative recent approaches have recently been attempted to increase the intention-to-treat (ITT) eradication rate, possibly over 85% (Table 1) [47]. For many years, a recommended alternative approach (Maastricht III Consensus and Guidelines of American College of Gastroenterology) has been the addition of PPIs to the old regimen comprising a bismuth salt, tetracycline, and MNZ [48]. A meta-analysis on triple versus bismuth-containing quadruple Therapy for HP infection demonstrated that 80% of patients who received quadruple therapy and 79% of patients who received triple therapy achieved eradication by ITT analysis, indicating a similar effectiveness for the two regimens, even though bismuth-based quadruple therapy is known to be useful after failure of PPI-based triple therapies (average eradication rate of 76% for quadruple therapy when used as second-line therapy) [49, 50]. 

An efficacy of ranitidine bismuth-based triple therapies has also been demonstrated as retreatments [51]. Non-bismuth quadruple therapy (PPI-CAM-AMPC-nitroimidazole) also represents a safe alternative to triple therapy and should be taken into consideration when the efficacy of triple therapy results in being unacceptably low [33]. A review article by Gisbert et al. [52] critically evaluated the evidence existing on the role of non-bismuth quadruple therapy (PPI-clarithromycin-amoxicillin-nitroimidazole) [53–55] in the treatment of HP infection. This regimen appears to be effective, safe, and suited for use in settings where the efficacy of triple therapy is unacceptably low. 

Because of the scarce compliance to these regimens as well as the increased resistance to both CAM and MNZ, the “sequential therapy” was introduced as a promising new treatment comprising 5 days of amoxicillin with a PPI, followed by 5 days of the same PPI but with a nitroimidazole (tinidazole, MNZ) together with CAM. The sequential therapy was largely experimented in Italy where an increase in the eradication rate by at least 10% was observed, compared with standard regimens, independently on clarithromycin resistance [56, 57]. This regimen has been demonstrated more effective than standard triple therapy by several clinical trials and meta-analyses [58, 59]. By the way, sequential therapy has not been subjected to any clinical trial in the United States, yet. A recent trial in South America, demonstrated the standard 14-day triple therapy to be more efficacious than the 10-day sequential four-drug regimen [60]. A study by Hsu et al. on a cohort of patients in Taiwan, compared an extended 14-day therapy to the 10-day sequential therapy, without attesting any improvement [61]. A study by Miehlke et al. showed that a high-dose PPI dual therapy with AMPC was effective as a salvage treatment for eradication of *H. pylori* resistant to both CAM and MNZ, with an ITT of 76% [62]. A study by Attumi and Graham evaluated the efficacy of a prolonged dual therapy (AMPC and omeprazole for 6 weeks) without attesting any improvement in treatment outcome (ITT of 63%)[63], thus suggesting that extending the duration of a dual therapy to more than 14 days is not effective for achieving an improved treatment outcome [47]. What finally emerges from this study is that higher doses and more effective agents represent a stronger approach for HP eradication than the simply longer duration [47]. The nonbismuth quadruple therapy and the sequential regimens resulted in being similar in terms of efficacy and safety. Several randomised controlled trials and meta-analyses have demonstrated that non-bismuth quadruple therapy is more effective and equally well tolerated as standard triple therapy. A meta-analysis of 15 studies showed a mean cure rate of 90% for non-bismuth quadruple therapy. CAM resistance may reduce the efficacy of non-bismuth quadruple therapy, although the eradication rate remains much higher than in standard triple therapy. Many studies have included Levofloxacin and shown good eradication rates, indicating a levofloxacin-based triple therapy as another option for patients with persistent infections [64].

In a recent trial by Basu et al. [65] a new regimen with high eradication rate was proposed: a novel drug combination was tested within a clinical trial and compared with a standard triple therapy (amoxicillin, CAM, and lansoprazole). This new regimen was called LOAD as it comprised three antibiotics (levofloxacin, doxycycline, and nitazoxanide, a prodrug with antiparasitic properties) together with omeprazole; its efficacy was supported by an eradication rate of 90% compared with 73% with the triple therapy. This relatively expensive novel drug combination would represent an absolute increase of 17% in the eradication rate (with a needed to treat number of 5.88 to achieve one more successful eradication). A concrete advantage of this regimen is the exclusion of any penicillin-based antibiotic, so that in those patients a real or perceived allergy to penicillin can be treated as well [7]. To be noticed as a limitation in the application of this regimen is the fluoroquinolone levofloxacin included in the treatment, which appears to rapidly generate resistance, with a peculiar increase recently demonstrated among Asian populations [9]. The elevated cost of nitazoxanide is also a socioeconomically issue to be take into consideration as regards the compliance to this new regimen proposed [7]. In Asia, the currently recommended first-line therapy for *H. pylori* infection is a PPI, AMPC, and CAM for 7 days [49]. According to the Maastricht III consensus, in naïve HP-infected patients, triple therapy using a PPI with CAM and AMPC or MNZ remains the recommended first-choice treatment [10]. Some authors have demonstrated the importance of *H. pylori* CagA status for the efficacy of antibiotic treatment considering an infection with cytotoxic strains as a good predictive marker of successful therapy [11–13, 66]. A useful role for some probiotics including *S. boulardii* and *L. reuteri* as an adjunct to HP eradication treatment has recently been established [67, 68]. 

## 6. Managing HP Infection in Children: The 2011 ESPGHAN and NASPGHAN Guidelines

In 2011 the European and North American Societies of Pediatric Gastroenterology Hepatology and Nutrition (ESPGHAN and NASPGHAN) emitted evidence-based guidelines for managing HP in children [69]. These guidelines suggest to assess not only the presence of the HP infection but also all the other possible underlying causes of the specific clinical features. They recommend to test children having first-degree relatives with gastric cancer as well as children showing refractory iron-deficiency anemia with previous negative tests for other causes. Instead, screening for HP is not recommended in children with suspect of functional abdominal pain. HP positive peptic ulcer disease (PUD) is recommended for treatment. However, for children with HP infection without PUD, treatment should be considered by the clinician evaluating the potential advantages and the risk factors of a developed chronic infection with bad consequences. In children, the first-line recommended therapies are

1. standard triple therapy with PPI (1-2 mg/kg/day), AMPC (50 mg/kg/day), CAM (20 mg/kg/day), or MNZ (20 mg/kg/day);
2. bismuth-based quadruple therapy: bismuth subsalicylate (8 mg/kg/day), AMPC (50 mg/kg/day), and MNZ (20 mg/kg/day);
3. sequential therapy: PPI (1-2 mg/kg/day) AMPC (50 mg/kg/day) for 5 days, then PPI (1-2 mg/kg/day), CAM (20 mg/kg/day), and MNZ (20 mg/kg/day) for 5 days.

As for adults, an antibiotic susceptibility test should be performed whenever a high resistance rate (>20%) in the area is known. Once treatment is completed a non invasive test should be done to ensure eradication [69].

## 7. *H. pylori* Infection and Diseases of the Upper Gastrointestinal Tract

Over the past two decades, HP has represented a pivotal role in nonmalignant diseases of the upper gastrointestinal tract, as peptic ulcer disease (PUD), functional dyspepsia (FD), and gastroesophageal reflux disease (GERD) [70]. Irrespective of etiology, both incidence and prevalence of uncomplicated PUD have dramatically decreased in the developed countries in the last decades. By the way, rates of both ulcer complications and mortality still remain high nowadays. The prevalence of HP-associated ulcers is decreasing, whereas a role of nonsteroidal anti-inflammatory drugs (NSAID) is becoming predominant. In fact, in western countries a decline of HP prevalence is described as well as an increasing use of NSAIDs and aspirin, the latter mainly prescribed for cardiovascular protection [70]. As HP-related PUD is the cause of symptoms in only a minority of patients with dyspepsia, recommendations on HP testing and subsequent eradication in all patients with dyspeptic symptoms vary greatly [71]. 

As regards FD (as defined by Rome III criteria) a possible role of HP still remains uncertain [69]. The pathophysiology of FD is actually multifactorial. Several randomized controlled trials performed in western countries and in Asia have given opposite results about the role of HP infection in causing FD, with no evidence for the former countries whereas a success of eradication has been obtained in some east Asian populations [70]. As HP may reduce gastric acid secretion, its colonisation has been reported to be inversely associated with the development of GERD [72]. HP infection would be protective towards gastroesophageal disease (GERD). By the way, HP is associated with esophageal squamous cell carcinoma, and long-term PPI treatments in patients with persistent HP infection may cause gastric atrophy, which is a precancerous lesion [72, 73]. It is reported that the widespread use of eradication therapy with a falling prevalence of HP infection has been paralleled with an increase in the occurrence of GERD [74]. By the way, other studies evaluating the same relation actually produced inconsistent results [75]. A systematic review by Qian et al. [76] was aimed to evaluate whether the eradication of HP would worsen or improve GERD, both from the clinical (heartburn, acid regurgitation) and endoscopical (erosive esophagitis) point of view. The 11 articles included in this met-analysis showed no significant difference between patients with HP eradicated and those with persistent infection. The authors therefore concluded that HP eradication does not worsen the clinical outcomes of GERD. Gastric cancer remains a major cause of cancer death worldwide [77]. Its pathogenesis is multifactorial and involves bacterial, host, and environmental factors that interplay in the histological progression to neoplasia. HP has been recognized as a major factor in the early stages of cancer development, so its screening and eradication are mandatory for cancer prevention. Population-based screening represents the current best option for the primary prevention of gastric cancer, the incidence of which largely depends on differences in HP CagA status and dietary factors [13, 66, 78]. To predict the risk of gastric cancer development and diagnose atrophic gastritis, new serologic testing for a combination of pepsinogen I and II and gastrin and HP antibodies has yielded satisfactory results over the last decades. Also old age, alcohol, and smoking habits are risk factors for gastric cancer. A prevention of gastric cancer can be obtained by eradication of HP, the success of which depends on the degree of preneoplastic changes (gastric atrophy and intestinal metaplasia) at the time of eradication. 

Virulence determinants of HP may be connected to gastric carcinogenesis; the use of genomewide germline analyses might offer the chance to identify high-risk groups of gastric cancer [79]. 

## 8. Conclusions

Many different factors play a role in the development of HP infection, including geography, ethnicity, age, and socioeconomic elements. The efficacy of various available treatment strategies varies significantly within different regions. A progressive decline in infection rates has been secondary to the improvements in sanitation as well as to the use of combination anti-HP regimens over the past decades. The gradual increase in failure of HP eradication treatments is mainly secondary to antimicrobial resistance, in particular to clarithromycin.

Quadruple therapy (PPI + CAM + AMPC and/or MNZ ± Bismuth) and sequential therapy (PPI + AMPC for 5 days, then PPI + CAM + MNZ for other 5 days) are efficacious alternatives to standard triple therapy for both adults and children. The main current effort should be to maintain the eradication rate of HP above 85% and to prevent any increase in antimicrobial resistance in the general population. The possibility of a tailored therapy will be a chance in the next future to optimize the most effective eradication regimen within the various geographical areas.

## Figures and Tables

**Table 1:** Summary of the main current first- and second-line treatment regimens available for HP eradication.

First-line treatment regimens		
Triple standard therapy	PPI + CAM + AMPC and/or MNZ	Eradication of HP infection from 90% to 70–80% Steadily decline in treatment efficacy in USA [7]
Sequential therapy	PPI + AMPC for 5 days, then PPI + CAM + MNZ for other 5 days	Eradication rates of 90%–94% [8, 9]
Concomitant therapy	PPI + CAM + AMPC + MNZ for 7–10 days	Eradication rate of 90%. More simple regimen, good alternative to standard triple therapy [10]
Bismuth-based quadruple therapy	PPI + Bismuth + Tetracycline + MNZ for 10–14 days	Important role in countries with high CAM resistance rate; in a recent study patients took PPI and a three-in-one capsule containing bismuth subcitrate potassium, MNZ and Tetracycline with eradication rates of 80% versus 55% in the standard therapy group [11]

Second-line treatment regimens		

Levofloxacin-based triple therapy	PPI, levofloxacin, and AMPC	Good alternative for patients who failed with standard treatment. A recent meta-analysis highlighted that levofloxacin-based triple therapy has lower incidence in side effects than the bismuth-based quadruple therapy, as well as a better eradication rate (87% versus 68%) [12]
Rifabutin-containing rescue therapy		Well tolerated, good alternative for patients who failed with a first-line therapy [13]

AMPC: amoxicillin, MNZ: metronidazole, CAM: clarithromycin, and PPI: proton pump inhibitor.
